# Deep sequencing of evolving pathogen populations: applications, errors, and bioinformatic solutions

**DOI:** 10.1186/2042-5783-4-1

**Published:** 2014-01-15

**Authors:** Kerensa McElroy, Torsten Thomas, Fabio Luciani

**Affiliations:** 1 Centre for Marine Bio-Innovation and School of Biotechnology and Biomolecular Sciences, UNSW, Sydney, NSW 2052, Australia; 2 Inflammation and Infection Research Group, Evolutionary Dynamics of Infectious Diseases, School of Medical Sciences, University of New South Wales, Sydney, NSW 2052, Australia

**Keywords:** Next generation sequencing, Deep sequencing, Virus genome, Bacterial genome, Evolution, Bioinformatics, Haplotype reconstruction, Statistical errors

## Abstract

Deep sequencing harnesses the high throughput nature of next generation sequencing technologies to generate population samples, treating information contained in individual reads as meaningful. Here, we review applications of deep sequencing to pathogen evolution. Pioneering deep sequencing studies from the virology literature are discussed, such as whole genome Roche-454 sequencing analyses of the dynamics of the rapidly mutating pathogens hepatitis C virus and HIV. Extension of the deep sequencing approach to bacterial populations is then discussed, including the impacts of emerging sequencing technologies. While it is clear that deep sequencing has unprecedented potential for assessing the genetic structure and evolutionary history of pathogen populations, bioinformatic challenges remain. We summarise current approaches to overcoming these challenges, in particular methods for detecting low frequency variants in the context of sequencing error and reconstructing individual haplotypes from short reads.

## Introduction

Next generation DNA sequencing (NGS) is characterised by extremely parallel, cost efficient sequencing of genomic fragments, generating hundreds of thousands to hundreds of millions of short ‘reads’ in a single run [1]. This has opened up new possibilities in the study of pathogen evolution, allowing researchers to use ‘deep sequencing’ to track genomic changes over time. NGS has many useful applications, ranging from measuring gene expression levels [2,3], to discovering rare viruses [4] or metagenomically profiling communities [5,6]. However for the purposes of this review, we limit our scope to applications of NGS where aligned reads are considered to be a population sample. In this definition of deep sequencing, reads aligning to a given genomic position are each assumed to originate from an individual replicon, revealing a snapshot of the population’s genetic diversity.

Deep sequencing studies typically feature a depth of several hundred to several thousand reads at any given position, with the information contained within individual reads being treated as meaningful. This differs from resequencing projects, where genomic sequences are generated from the consensus of all aligned reads at any given position, and then used for comparative analysis with other genomes. Resequencing projects rarely exceed a read depth of around 100×, with excess depth being used to correct for sequencing errors [7]. For haploid genomes this ensures a correct estimate of the consensus sequence, as long as sequencing errors occur in less than 50% of reads at any position. This is in contrast to the deep sequencing approach, where variants with extremely low population frequencies (under 1%) may be of interest. As current NGS technologies have error rates within this order of magnitude, distinguishing true variants from errors is a key challenge of deep sequencing projects.

In this review, we first highlight current applications of deep sequencing to viral pathogens. Extension of the deep sequencing approach to bacterial populations is then considered, including unique bioinformatic challenges and the implications of emerging sequencing technologies. The second half of our review provides a detailed discussion of sources of errors in deep sequencing and ways of minimising their impacts, including computational approaches to identifying variants within the context of sequencing errors.

## Applications of deep sequencing

### Viruses

Virology has pioneered the deep sequencing approach, serendipitously combining the small genome size and fast evolution of viruses with the extremely parallel nature of NGS. This has led to the development of novel data analysis algorithms and pipelines, as well as significantly advancing our understanding of viral pathogens.

Table 1 describes a selection of viral deep sequencing studies, illustrating a variety of applications and approaches. In particular, deep sequencing has transformed the study of rapidly mutating RNA viruses, such as the human pathogens human immunodeficiency virus (HIV) and hepatitis C virus (HCV). These viruses experience error prone genome replication due to a lack of proofreading capacity, thus generating great diversity compared to other viral pathogens even within a single infection [8,9]. Deep sequencing is proving invaluable for studying this diversity and its evolutionary and clinical consequences.

**Table 1:** Representative examples of deep sequencing applied to viral populations

**Pathogen**	**Design**	**Technology**	**Ref seq**	**Filter**	**Align**	**SNV**	**Hap**	**Application**	**Reference**
HIV	RT-PCR, nested PCR of pol fragment	Roche-454 GS-FLX amplicon sequencing	Sanger sequenced pol gene	In-house software: removes reads with ambiguous bases, < 80% similarity to reference, or outside region of interest	GS amplicon software (Roche, Penzberg, Germany), Needleman-Wunsch	In house scripts, manual inspection: remove gaps, remove reads with frameshift indels or stop codons, remove variants only contained in reads in one direction, positional variant cut-off values based on control sequences	Individual reads (40 bp region of interest)	Longitudinal emergence of drug resistance during treatment failure	[10]
HIV	RT, PCR amplificatin of 4 fragments (3.5 kb each). Full genome analysis	Roche-454 GS-FLX Titanium	*De novo* assembled reference using AssembleViral454 v1.0	NS	Mosaik	RC454 / V-Phaser	V-Phaser (one read length only)	Longitudinal emergence of CD8+ T cell escape variants, viral adaptation	[11]
HCV	RT, PCR amplification of HVR-1, nested PCR using sequencing adapters	Roche-454 GS-FLX Titanium amplicon sequencing	358 HCV HVR-1 representative sequences from Los Alamos National Laboratory HCV	Flow clustering as implemented in QIIME, only reads covering entire region of interest	MAFFT (multiple sequence alignment)	NA	Individual reads	Identification of a transmission event	[12]
HCV	Whole-genome library prep direct from RNA isolated from human serum, using mRNA-seq sample prep kit (Illumina, San Diego, CA)	Illumina GA IIx 76 bp single end reads	970 reference HCV sequences registered at the Hepatitis Virus Database server	Primer stripping using CLC Genomics Workbench (4.6), remove reads aligning to human genome, removal of duplicate reads	BWA 0.5.9-r16	Samtools (0.1.16)	NA	PCR-free whole genome HCV sequencing from human serum; variant comparison between treatment naïve and treatment experienced patients	[13]
HCV	RT-PCR using genotype specific primers, nested PCR of full genome, followed by random shearing and library preparation	Roche-454 GS-FLX Titanium	Sanger-sequenced consensus	In house software (discard reads with Phred quality scores below 20 or length < 55nt)	Mosaik	ShoRAH, manual cleaning	ShoRAH (up to 1600 bp reconstructions)	Within-host evolution/genetic bottleneck	[14]
HRV	Duplicate whole-genome RT-PCR of overlapping primer pairs, nebulisation of pooled fragments and library prep	Illumina GA IIx	Sanger-sequenced consensus	Illumina software: RTA SCS.2.6 and CASAVA 1.6	MAQ v0.7.1	In house scripts; cut-off based on statistical analyses of base frequencies along reference. Comparison between replicates.	NA	Within-host evolution during immunosuppression	[15]
		76 bp single end reads							
Dengue	RT, PCR amplification of four different fragments, random shearing and adapter ligation	Roche-454 GS-FLX Titanium	*De novo* assembled using AV454 with manual finishing	NS	Mosaik	RC454/ V-Phaser. Manual removal of variants in primer binding sites or only in ends of reads	NA	Intra-host viral diversity	[16]
Poliovirus	RT-PCR and nested PCR of target amplicon, followed by random shearing and library preparation	Roche-454 FLX Titanium and Illumina GA IIx 76 bp single end reads	Known amplicon sequences	Proprietary Roche/Illumina software. In house software (discard reads with Phred quality scores below 20).	NS	Custom made scripts – disregard variants with strand bias, as well as insertions and deletions adjacent to homopolymers for Roche-454 data.	NA	Detection of emerging resistant variants in a vaccine stock	[17]

Details of the experimental design and analysis pipeline for various applications of deep sequencing to different viruses are given. ‘Design’ describes the types of samples used and any sample processing up to library preparation. ‘Technology’ indicates the type of sequencing employed. ‘Filter’ details any pre-alignment read processing steps. ‘Ref. Seq.’ describes what kinds of reference sequences were used for read alignment, while ‘Align’ gives the actual alignment software used. ‘SNV’ and ‘Hap.’ indicate software used for SNV detection and haplotype reconstruction respectively. ‘Application’ describes the biological motivation for the study. ‘NS’ indicates the method was not specified in the cited publication, while ‘NA’ means not attempted.

As an example, deep sequencing has been used to comprehensively characterise within-host evolution of HCV during the early acute phase of infection. This has shown that transmission of virions to a new host represents a genetic bottleneck, with four or fewer viral variants successfully initiating new infections [18]. Bull *et al.* extended this approach by phylogenetically analysing longitudinal whole genome HCV deep sequencing data. This study demonstrated that acute infection is also characterised by a second genetic bottleneck, which occurs at 100 days post infection, regardless of infection outcome (either viral clearance and recovery, or chronic infection characterised by emergence of a new viral variant) [14]. Longitudinal whole genome deep sequencing has also been used to characterise acute HIV-1 infections. For example, deep sequencing was applied to detect the rapid emergence of low frequency ‘escape’ variants, arising as adaptations to host CD8+ T-cell responses [11].

This ability to detect low frequency variants is an important feature of deep sequencing, for example in the context of drug resistance. Prior to the advent of deep sequencing, screening for resistance to antiviral drugs typically involved amplifying and sequencing a single PCR product from a population of viral particles in a plasma sample, followed by manually scanning the chromatogram for minor peaks. This method has a detection threshold corresponding to a variant population frequency of around 20% [19]. Recently, deep sequencing has been shown to allow the detection of protease inhibitor resistance mutations in HIV with population frequencies < 1% [20]. Several other studies have used deep sequencing to detect low frequency drug resistance mutations in hepatitis B virus (HBV) [21], HCV [22], and influenza virus [9].

In addition to providing insights into within-host evolution, deep sequencing of samples from populations of infected individuals can be used epidemiologically to dissect transmission events. For example, a recent deep sequencing study demonstrated HCV transmission between injecting drug users in Mexico [12], while a detailed deep sequencing analysis of norovirus transmission within a household showed that rare variants may survive transmission, with chronic infections providing a reservoir of new viral variants [23].

### Bacteria

To date, NGS of bacterial pathogens has focussed largely on resequencing of individual isolates, rather than deep sequencing of populations. For example Saunders *et al.* followed within-host evolution of *Mycobacterium tuberculosis* by sequencing isolates collected from a patient undergoing treatment over a period of 12 months [24]. Only two point mutations were found, occurring sequentially and conferring resistance to isoniazid and rifampicin, respectively. The authors argue that the conspicuous lack of other mutations implies a low per base mutation rate. However, resequencing of isolates only allows identification of mutations that have or are close to reaching fixation. It is therefore unsurprising that the identified mutations confer antibiotic resistance, most likely arising due to selective sweeps induced by the treatment regime. A deep sequencing analysis of the full mutation spectrum within each isolate has the potential to give a more complete view of the evolutionary dynamics of the infecting population.

A recent study of *Staphylococcus aureus* dynamics used an approach of individual colony sequencing, performing whole genome resequencing of multiple colonies derived from individual isolates [25]. Isolates were harvested at several time points, for two nasal carriers and one patient who progressed from nasal carriage to fatal bloodstream infection. Whilst not using individual reads as a population sample, this study embodies many of the concepts behind deep sequencing. By phylogenetically analysing the pattern of identified variants, the evolutionary history of infection was reconstructed.

Resequencing a selection of colonies from individual isolates cannot, however, match the sample depth obtained through deep sequencing a population. Sequencing of populations, on the other hand, has the drawback that unless variants co-occur within a read length or read pair, it is difficult to reconstruct individual haplotypes and perform phylogenetic analysis. A lack of physical linkage of variants within reads is more problematic for bacteria than viruses, due to their relatively larger genome size, smaller per base mutation rates, and slower rates of evolution. However, NGS reads are continuously increasing in length (Roche-454 read lengths are now approaching 700 nt, while Pacific Biosciences RS reads may be up to 15000 nt) (Table 2), making reconstruction of individual bacterial haplotypes from deep sequencing data realistic in the near future.

**Table 2:** Sequencing technologies, features and errors

**Platform**	**Manufacturer**	**Throughput (per machine run)**	**Reported errors**	**Depth (virus)**	**Depth (bacteria)**	**Reference**
454 GS Junior	Roche	~135 K reads @ ~520 nt	~0.38% indels	7 K	14	[26]
GS-FLX Titanium	Roche	~1 M reads @ ~500 nt	~0.28% indels; ~0.12% substitution (max 1.07%)	50 K	100	[27]
MiSeq	Illumina	~ 11 M reads @ ~ 150 nt	< 0.001% indels, ~0.1% substitutions	165 K	330	[26]
GA IIx	Illumina	~ 640 M reads @ 100 nt	~0.001% indels; ~0.31% substitutions (max ~5.85%)	6 M	13 K	[27]
HiSeq 2000	Illumina	~ 6G reads @ 100 nt	~0.002% indels; ~0.32% substitutions (max ~8.2%)	60 M	120 K	*
Ion Torrent PGM	Life technologies	~2 M reads @ ~121 nt	~1.5% indels	24 K	48	[26]
SOLiD	Life technologies	~120 M reads @ ~50 nt	~0.09% substitutions (max > 5%)	600 K	1 K	[28,29]
RS	Pacific biosystems	~200 K reads @ ~2000 nt (max > 15000 nt)	~14% indels, ~1% substitutions	40 K	80	[30,31]
tSMS	Helicos	~1G reads @ 35 nt	~3% indels, ~0.2% substitutions	3 M	7 K	[32]

Indels errors are largely associated with homopolymers for Roche and Ion Torrent. This fact can have a significant impact on the detection of variants associated with homopolymers, as was recently shown for the 2184delA mutation of the cystic fibrosis transmembrane conductance regulator (CFTR) using Ion Torrent PGM [33]. Sequencing errors are also highly dependent on the sequencing context and thus can influence variant calling in a biased, but potentially predictable way. For example, certain GC-rich motifs have been reported to have substitution errors of close to 6% [27] for the Illumina sequencing technology. Depth columns give anticipated read depth for a typical viral (~10 K) or bacterial (~5 M) genome.

*Calculated for this review from control PhiX data using GemSIM v1.6 [27].

In a move towards deep sequencing, early studies have attempted to use individual sequencing reads to reveal the structure of bacterial populations. In a 2008 study of an acid mine drainage biofilm, Tyson *et al.* were able to reconstruct several *Leptospirillum* group II substrains [34]. Although this analysis was facilitated by the use of relatively long (~700 nt), low error, paired end Sanger sequenced reads obtained from a shotgun plasmid library, some modern NGS technologies already have similar features (Table 2). In fact, a study using Roche-454 sequencing to longitudinally investigate gut microbiota during the first three weeks of a premature infant’s life was able to reconstruct two closely related *Citrobacter* UC1CIT strains [35]. Comparative genomic analyses of these two strains highlighted regulatory, metabolic, and host interaction traits as possible drivers of early ecological differentiation.

The acid mine drainage and gut microbiota projects presented above featured a read depth of only 20× and 13×, respectively (i.e. a relative low depth sequencing effort). Due to the limited read depth of these studies, fast read alignment was not critical. However, while rapid algorithms for short read alignment have been developed, alignment of longer NGS reads currently represents a bottleneck, requiring faster algorithms [36]. Also, in the acid mine drainage and gut microbiota studies [35] much of the sub-strain demarcation was performed manually. Although work has been done on automating the analysis of deep sequencing data for viral populations, the larger genome size and lower levels of diversity for bacteria will require the development of novel algorithms. Interesting recent developments include programs to calculate the scaled mutation rate and the recombination rate of bacterial populations from the information contained in individual sequencing reads [37,38]. To the best of our knowledge, however, these programs have only been used on data with read depths of under 100×. Approaches developed for the analyses of deep sequencing data from cancer samples may prove useful for bacterial populations, as the evolution of cancer and the within-host evolution of bacterial pathogens share many features, including expanding, asexually reproducing clonal population structures, and the propensity for drug resistance [39]. For instance, the matched samples approach discussed below could be readily applied to longitudinal bacterial samples.

Very recently, two studies have used NGS deep sequencing on the level of entire bacterial populations (see Table 3). Firstly, deep sequencing was used for the sensitive detection of drug resistance mutations in bacteria, similar to what has already been performed for viruses (see above). Daum *et al.* employed the Ion Torrent platform to characterise five full length *Mycobacterium tuberculosis* genes (cumulatively sequencing 11.4 kb per isolate to a depth of 300-500×), in order to determine drug resistance in MDR and XDR strains and to discover heterogeneous or mixed strain genetic populations within isolates [40].

**Table 3:** Studies applying deep sequencing to within-population bacterial variation

**Pathogen**	**Design**	**Technology**	**Ref seq**	**Filter**	**Align**	**SNV**	**Hap**	**Application**	**Reference**
*M. tuber- culosis*	Chemical shearing of pooled PCR-amplified target genes (*rpoB*, *katG*, *pncA*, *gyrA, rrs*) for each isolate, followed by adapter ligation, barcoding, PCR amplification, and library preparation	Ion Torrent 314 PGM, generating 60-70 bp reads at 300-500×	NS	NS	NS	NS	NA	Detection of low-frequency drug resistance mutations	[40]
*S. aureus*	Extraction of genomic DNA followed by whole genome standard SOLiD mate-pair library construction, with 3 kb fragment size	SOLiD 3 plus, 2 times 50 bp reads at ~5000×	*S. aureus* SA957	SOCS package: quality threshold of Q15 and trimming to 42 bp	SOCS package	Detect and filter using SOCS package (min. av. qual 20, 500 < read depth < 15000, apply Bernoulli test (*p* < 0.001) to remaining SNVs	NA	Genome evolution	[41]

Details of the experimental design and analysis pipeline for the two examples of deep sequencing applied to bacterial populations identified in this review. ‘Design’ describes the types of samples used and any sample processing up to library preparation. ‘Technology’ indicates the type of sequencing employed. ‘Filter’ details any pre-alignment read processing steps. ‘Ref. Seq.’ describes what kinds of reference sequences were used for read alignment, while ‘Align’ gives the actual alignment software used. ‘SNV’ and ‘Hap.’ indicate software used for SNV detection and haplotype reconstruction respectively. ‘Application’ describes the biological motivation for the study. ‘NS’ indicates the method was not specified in the cited publication, while ‘NA’ means not attempted.

Secondly, a recent paper used deep sequencing to investigate mutations arising in a *Staphylococcus aureus* SA957 strain culture grown in Luria-Bertani broth and harvested during the late logarithmic growth phase. Whole genome deep sequencing (median depth of over 5,000×) combined with phylogenetic inference of ancestral sequences revealed that patterns of mutations were completely different from those obtained by standard comparison of closely related *S. aureus* strains [41]. For instance, there were significant differences in the distribution of both coding and non-coding SNVs, as well as in the location and frequency of indels. This finding questions the assumption that comparing the consensus genomes of individual strains is a good proxy for studying short term evolution. It also proves that deep sequencing of bacterial populations is technically possible, and has the power to reveal important aspects of bacterial evolution that may previously have been missed by shallow sequencing of individual strains or isolates.

## Sources of error in deep sequencing

The examples highlighted above demonstrate that deep sequencing is a powerful tool for investigating evolving viral and bacterial populations, facilitating elucidation of evolutionary dynamics, detection of drug resistance and immune escape mutations, and characterisation of transmission networks. Key to all of these applications is the accurate identification of genetic variants. At its most basic level, this means accurately quantifying the population frequency of individual point mutations, otherwise known as single nucleotide variants (SNVs). Some applications, for instance phylogenetic analysis, also require the pattern of co-occurrence of SNVs within subsets of the population to be determined; either by simply considering physical linkage of SNVs within individual reads, or by using overlapping reads to reconstruct longer genome fragments, termed ‘haplotypes’. The accuracy of both SNV detection and haplotype reconstruction is jeopardised by errors occurring during deep sequencing.

**Figure 1:**
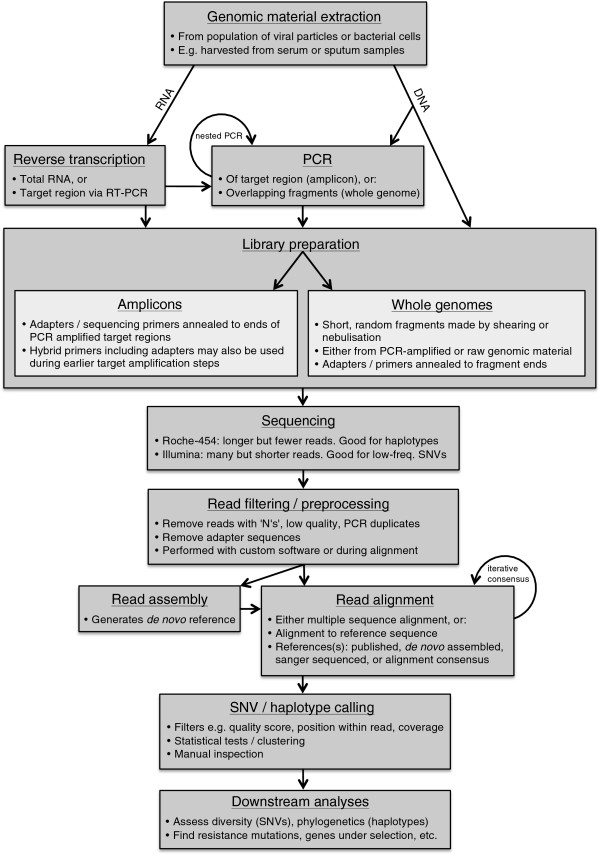
**Flowchart detailing pipeline steps required for deep sequencing projects.** After extracting genomic material, PCR amplification may be required prior to library preparation. For sequencing of a target region (‘amplicon sequencing’), multiple, ‘nested’ PCR rounds may be performed. Sequencing adapters and primers may be included in the primer for the final round, or may be annealed to the ends of fragments after amplification. For whole genome sequencing, multiple, overlapping PCR products are randomly sheared before annealing of sequencing adapters and primers. Alternatively, if sufficient genomic material is available, shearing and annealing may be performed directly without PCR amplification. If sequencing RNA, RT must be performed before library preparation. For amplicon sequencing, this may take the form of an initial RT-PCR. Choice of sequencing technology is dependent on the project’s aims: for instance, the longer reads of Roche-454 may be more appropriate for reconstructing haplotypes, while the high data volume afforded by Illumina is more suitable for detecting very low frequency SNVs. After sequencing, reads must be aligned, either via multiple sequence alignment or to a reference. Choice of reference is critical; if available, a published reference or references may be used; alternatively, a consensus sequence may be used, generated through *de novo* assembly, or by alignment to a published reference followed by replacement of fixed variants, or by Sanger sequencing the same sample as submitted for deep sequencing. Following alignment, a number of bioinformatic tools are available for SNV calling, haplotype reconstruction, and downstream analysis.

Errors may occur at any of the many steps involved in deep sequencing an evolving pathogen population, including during DNA or RNA extraction; reverse transcription for RNA viruses; PCR amplification of target regions; library preparation and sequencing; read quality control and filtering; *de novo* assembly, consensus calling, and alignment; variant calling and haplotype reconstruction; and further downstream analysis of results (Figure 1). The following sections discuss sources of error during the various steps in a deep sequencing pipeline, and ways of minimising these errors through careful experimental design and data analysis. Our discussion is mainly limited to NGS data produced by the Roche-454 and Illumina platforms. These have been the most popular technologies for deep sequencing in recent times; their errors are well understood and methods to correct such errors have been developed. However, we believe many of the general insights into sequencing errors, and bioinformatic solutions for dealing with them, will also be broadly applicable to emerging technologies.

### Sample collection and PCR errors

Firstly, genomic material must be obtained from the sample. For heterogeneous community samples (metagenomes), choice of DNA extraction method can significantly affect the representation of individual members [42,43]. Fortunately, this is likely to be less problematic for deep sequencing of single species populations, which generally consist of closely related individuals that will have similar properties with respect to cell lysis and release of genomic material. However, particularly in the case of viruses with low titres or sample amount, nucleic acid yield may be insufficient, requiring PCR amplification prior to sequencing. For whole virus sequencing, overlapping fragments may be amplified along the length of the genome, before random shearing and ligation of sequencing adapters [11,14]. For RNA viruses such as HCV and HIV, reverse-transcriptase (RT) PCR may be applied [14,15]. Alternatively, RT may be performed independently before regular PCR amplification [16]. If whole genome sequencing is not required, smaller target regions can be amplified directly or by using multiple, ‘nested’ rounds of PCR. Hybrid primers with integrated sequencing adapters are often used for the final round (i.e., ‘amplicon sequencing’) [12].

PCR amplification, either prior to sequencing or during library preparation, can introduce both biases and errors. Differences in primer binding affinities between templates, and re-sampling of individual templates (i.e., ‘PCR duplicates’) can result in amplification bias, distorting variant frequencies. Measurement of amplification bias using uniquely tagged primer sequences to infer the template of origin of each sequence read found that observed frequencies typically differed by two to 15 fold compared to true frequencies. In some cases, the bias was up to 100 fold [44]. Chimeras can also form during the PCR reaction, leading to the artificial creation of non-existent templates. In a deep sequencing analysis of a mixture of HIV clones, the estimated PCR chimera rate was 1.9% [45]. Estimates of PCR chimera rates from studies not employing deep sequencing vary from 1% to 5%, depending on the length of elongation time used [46]. Additionally, the polymerases used during PCR have their own inherent error rates, introducing mistakes that can mimic true variants. A polymerase error rate of 0.2% has been inferred from the HIV mixture study described above [45]. Other measures of polymerase error rates range from 10^-3^ to 10^-6^[47].

Even when enough genomic material can be obtained to allow direct whole genome sequencing, NGS library preparation typically still involves a PCR step. For both Roche-454 and Illumina sequencing, DNA is first subjected to random shearing, followed by isolation of suitably sized fragments. During this step, chimeras between sheared fragments may form. After shearing and fragment size selection, DNA is then ligated to oligonucleotide adapters, which are used to immobilise fragments and to provide primer binding sites for clonal amplification. In this latter step, errors may be introduced due to PCR amplification. Small sections of adapter sequences may also be retained in some reads due to adapter mediated recombination, appearing as indels in aligned data [27].

### Sequencing errors

Errors are also introduced during synthesis of actual DNA sequencing reads. Table 2 gives estimates of error rates for several NGS platforms. Roche-454 and Illumina sequencing errors have been studied in some depth. Originally, Roche-454 error rates were estimated at 4% for experimental samples, and 0.6% for test fragments [48]. Margulies *et al.* explained this discrepancy by suggesting that in sequencing libraries from experimental samples, some ‘clonally’ amplified fragments may in fact not be clonal, originating from two or more fragments and thereby inflating errors [48]. Subsequent versions of Roche-454 have greatly improved on these error rates, with, for example, Huse *et al.* estimating error rates for the GS20 sequencer at 0.49% for experimental data, and at an even lower 0.1% for test fragments [49]. Roche-454 test fragments do not undergo library preparation or PCR amplification before sequencing, explaining their lower error rates. In fact, one study used this feature of Roche-454 test fragments to perform an in depth analysis of errors occurring during actual read synthesis on the GS-FLX Titanium platform [50]. Error rates were found to be highly heterogeneous, with an average error rate of 1.07% and a local maximum error rate of over 50% in some cases [50]. The presence of homopolymers, sequence position, read length, and spatial location within the PicoTitre plate were identified as factors influencing local error rates [50]. Indel errors associated with homopolymers are widely recognised as the main source of error in Roche-454 sequencing and there is emerging evidence that Ion Torrent sequencing also suffers from the same type of errors [33]. Indel errors can have a significant impact on the detection of variants associated with homopolymers, as was recently shown in Ion Torrent PGM screening for cystic fibrosis transmembrane conductance regulator (CFTR) mutations. The 2184delA mutation, located within a 7 bp homopolymer, was the only studied mutation not able to be reliably detected [33].

Error rates are also known to be heterogeneous for Illumina sequencing. Local sequence context, including GGC sequences, inverted repeats, and homopolymers are all known to inflate downstream error rates [27,51]. For example, GC-rich motives have been reported to suffer from substitution error rates close to 6% [27]. Folding of single stranded DNA, and sequence specific effects on the activity of DNA polymerase (e.g. slippage or stalling), have been proposed as underlying mechanisms [51]. Average error rates have been estimated at between 0.31% and 1.66%, varying between individual sequencing runs [52], even when performed on the same machine [27]. Error rates also increase along the length of a read, in some cases being ten fold higher at the 3′ end than the 5′ end [52]. Roche-454 error rates have also been observed to increase slightly along the length of a read [27]. In fact, the higher error rate reported for GS-FLX Titanium platform reads (~350 nt) compared to GS20 reads (~150 nt) can be explained by a higher error rate towards the end of longer reads. This is further illustrated by the fact that for GS-FLX Titanium reads, the error rate for experimental data fell to 0.53% when only the first 101 nucleotides of a read were considered [50].

### Alignment errors

Alignment of sequencing reads can itself introduce bias and errors. Aligning to a known reference can be attractive when the aim of a study is to compare samples, as, for example, in the longitudinal analysis of within-host HCV evolution [14]. However, use of an inappropriate (i.e. too distantly related) reference can lead to spurious alignment or alignment failure. One way to overcome this issue is to generate a consensus sequence. This may be done either through iterative alignment to a published reference, with replacement of any fixed SNVs between rounds, or by Sanger sequencing the same sample as used for deep sequencing, as in [10] (only possible for small genomes or amplicons). Even still, if subsets of the sequenced population diverge too much from the consensus sequence, reads originating from these sub-populations may not align, thus biasing results. Choice of alignment algorithm is critical in overcoming this problem and needs to be carefully considered. For instance, the original version of the Bowtie algorithm [53] did not allow for alignment with gaps. For reads with homopolymer errors, this results in either alignment failure or long stretches of mismatches. As another example, SOAP2 does not allow more than two mismatches in aligned reads [54]. Under this condition, data from rapidly evolving viruses such as HIV or HCV, or long reads where more than two errors are expected, may not align.

### Haplotype reconstruction errors

Haplotype reconstruction involves both collapsing individual, error prone reads into their source haplotypes, and assembling longer genomic segments from these short, overlapping read-length haplotypes. The amplification bias and chimera errors discussed above negatively impact the accuracy of reconstructing read-length haplotypes. When reconstructing longer haplotypes through assembly, *in silico* chimeras may also be artificially generated. For instance, the program ShoRAH has been shown to reconstruct read-length haplotypes with population frequencies as low as 0.1% in control data [45]. When the reconstruction of longer haplotypes was attempted on real HCV data, the comparison with cloned haplotype sequences demonstrated a detection limit of > 2.5% due to the formation of low frequency *in silico* chimeras [14]. *In silico* chimeras can occur when multiple connecting paths through the overlapping read length haplotypes are possible. Single nucleotide errors (point errors) can confound this problem, by creating misleading paths, leading to more chimeras and also inflating the number of non-chimeric haplotypes identified. In deep sequencing studies of pathogen evolution, these problems complicate both recombination detection and phylogenetic analysis of haplotypes, and also inflate diversity estimates.

## Overcoming errors

### Sample collection and PCR

While sample collection and PCR biases may be difficult to eliminate, they are often systematic, which at least for comparative analyses may limit their impact provided experiments are appropriately designed. For DNA extraction, it has been shown that technical replicates of the same method vary less than different extraction methods, even when performed by different experimenters on different days [43]. The best way to reduce PCR biases is to limit the use of PCR. Emerging single molecule sequencing technologies, such as Pacific Bioscience’s RS [31] platform and Oxford Nanopore Techonolgies’ GridION, have great potential here as they don’t employ PCR [55]. The RS platform involves immobilising individual DNA molecules at the bottom of ‘zero mode waveguide’ (ZMW) structures. Each ZMW acts as a miniature light microscope, recording the incorporation of individual fluorescently labelled nucleotides during elongation by a polymerase. With thousands of ZMWs on each sequencing cell, massively parallel single molecule sequencing is achieved. Oxford Nanopore Technologies take a different approach; a voltage is applied across an electrically resistant membrane with an embedded nanopore, causing individual DNA or RNA molecules to pass through the nanopore. As the molecules pass, individual nucleotides make characteristic disturbances to the current across the nanopore, allowing the sequence to be deduced. Sensor chips record from multiple nanopores simultaneously, facilitating parallel single molecule sequencing. Although single molecule sequencing may represent the future of deep sequencing, its usefulness in population genomics cannot currently be fully assessed. GridION is not yet commercially available and hence has not received independent error evaluation, and RS still has reported error rates in excess of 15% [30,31], which will substantially reduce the accuracy and sensitivity of variant calling. Innovative experimental designs, including for instance circular sequencing where the same DNA molecule is sequenced multiple times to generate a single sequence consensus [56], may help overcome these high error rates and thus improve the usefulness of single molecule sequencing for population studies.

In a novel approach, PCR free Illumina based HCV deep sequencing was achieved by sequencing the total RNA extracted from human serum samples and then discarding any reads aligning to the human genome before analysing the remaining reads. This approach achieved an average read depth of more than 50×, covering more than 99% of the HCV genome [13]. Greater depth using similar approaches could now be achieved with the Illumina HiSeq platform (Table 2), as recently proposed for direct RNA sequencing of HIV and other RNA viruses [57]. Although bias may still be introduced during the preparation of RNA samples, direct sequencing eliminates biases and errors introduced during the RT step and subsequent PCR amplification, providing a more accurate population sample, especially for low frequency variants.

PCR free sequencing is often not possible, however. The approach discussed above relies on very high volume data; for applications requiring Roche-454′s superior read length, the generation of the required amount of data may be prohibitively expensive (less data is produced overall by Roche-454 sequencing compared to Illumina sequencing, despite its longer read length (Table 2)). PCR amplification biases appear to be fairly reproducible for a specific primer pair [58]. Therefore, if the aim of a study is to compare samples, the effects of bias may be minimised through consistent experimental design. Alternatively, if PCR bias is likely to have a significant and unavoidable impact on data interpretation, biases may be accounted for using a primer tag system as demonstrated in a deep sequencing analysis of the HIV-1 protease gene [44]. In this study, random tags unique to individual primers were integrated in the primers of the first amplification round. This meant that even though PCR biases occurred, the fragment from which each sequenced product originated could be tracked, allowing bias to be both measured and accounted for before estimation of variant frequencies.

### Read filtering and alignment

Filtering reads prior to or during alignment is often performed in an attempt to reduce sequencing errors. By simply removing reads containing unspecified nucleotides from GS20 data, the average error rate was reduced from 4.7% to 0.24% [49]. Various programs are available for assessing read quality and filtering reads, for example BIGpre [59] and AmpliconNoise [60]. These tools also contain algorithms for removing PCR duplicates. The advantage obtained by filtering may vary with individual data set and sequencing platform. For example, one study found that 82% of Roche GS20 reads contained no errors, while for the Roche-454 GS-FLX platforms, only 10.09% of reads were without errors [49]. For Roche-454 GS-FLX datasets the reduction in error achieved through filtering of reads may be small compared to the cost in terms of coverage and depth. The impact on depth can be minimised by only trimming the error prone ends of reads, although this will reduce average read length. For instance, programs such as ConDeTri [61] and SolexaQA [62] trim the 3′ end of Illumina reads according to quality scores assigned to individual bases. Trimming may also be used to remove adapter sequences, for instance as implemented in the alignment program Novoalign (http://www.novocraft.com/).

Alignment biases can be easily detected by considering the proportion of aligned reads. For example, if a substantial fraction of reads remain unaligned, or the proportion of aligned reads varies between samples, then a different approach to alignment should be considered. A way forward is to align to a collection of related references, for instance from a public sequence database of a species [12]. Assembling the reference *de novo* before aligning reads back to the new reference(s), as in [11], may also help overcome these issues. While this is arguably the most accurate strategy, it can limit comparison between samples as each experiment or sample may end up with a unique reference sequence. Finally, both assembly and alignment are error prone, and the ‘best’ alignment chosen by an alignment program is not necessarily the ‘true’ alignment. Appropriate approaches and choice of assembly and alignment software will depend on the individual data set, experimental design, and aims (see [36,63] for a review and assessment of alignment software and algorithms). Alignment quality should also be inspected manually in a visualisation program such as Tablet [64].

### SNV detection and haplotype reconstruction

Whether the aim is simply to generate a list of SNVs, or to perform in an depth analysis of reconstructed haplotypes, the culmination of any deep sequencing pipeline involves identifying variants from aligned reads. Various programs offer solutions for calling SNVs, employing either cut-off based filtering or statistical tests to distinguish true variants from errors. Automated filtering of potential variants based on quality scores is a popular approach, as implemented in the program VarScan [65]. VarScan uses several criteria, including variant coverage and average variant base quality, to identify true variants. This approach assumes that errors within reads have correspondingly low quality scores. However, this assumption may be invalid in some instances. For example, errors occurring during PCR may be ‘correctly’ sequenced with high quality base calls. Also, for Roche-454 data, quality scores are not a direct per base indication of error probability, but rather an estimate of the confidence in the homopolymer length. Thus a genomic position within a longer homopolymer will generally be covered by reads with lower quality bases compared to a genomic position flanked by contrasting bases, independent of whether the individual read bases are correct or not [49]. For Illumina data, quality scores have also been shown to underestimate true error rates for high quality bases, and overestimate error rates for low quality bases [52]. Choosing appropriate cut-offs for quality score based variant calling is therefore difficult, making cut-off methods very sensitive to parameter choice [27].

An alternative, quality score independent approach is implemented in the program ShoRAH [45]. ShoRAH is essentially a haplotype reconstruction program; however any program that results in reconstructed haplotypes with associated frequency estimates can be used to call SNVs, by parsing the reconstructed haplotypes for variant sites (in fact, a list of SNVs is produced by ShoRAH as part of the standard output [66]). ShoRAH corrects errors by using Bayesian clustering to group reads into short haplotypes [67]. The consensus sequence within a group is then taken as the truth, with any deviations from the consensus removed as errors. A minimal set of haplotypes required to explain the error-corrected reads is constructed using parsimony methods, resulting in reconstructed long haplotypes. ShoRAH has been used to reconstruct long (1000 bp) HCV haplotypes from patient isolates, facilitating coalescent phylogenetic analysis of HCV evolution [14].

The program V-phaser uses a related approach, considering the ‘phasing’ or co-occurrence of variants within a read [68]. Additionally, V-phaser recalibrates individual base quality scores, incorporating both phasing and quality scores into its model of variant calling. Validation of this method by deep sequencing an artificial control mixture of West Nile virus samples demonstrated accurate detection of SNVs with population frequencies > 0.2% (sensitivity and specificity > 97%). Another study, employing a combinatorial model to reconstruct haplotypes from overlapping reads, detected 11 true haplotypes from deep sequencing data originating from a pooled sample of 12 HBV genomes [69].

Probabilistic methods like the examples given above assume that errors are random and that true SNVs are subject to selection and may therefore co-occur within a haplotype more frequently than is expected by chance. Under these assumptions, the SNV detection limit via clustering is a product of the per site error rate, the SNV frequency, and the number of physically linked polymorphic sites under consideration. By considering multiple variant sites within a read at once, increased statistical power can be achieved (see [66] for a formal mathematical argument). However, any systematic errors that co-occur within reads will violate the assumptions outlined above and behave like polymorphic sites, causing them to be retained during clustering or phasing analysis.

Statistical tests incorporating analysis of strand bias are emerging as one way of accounting for systematic sequencing errors [70]. The basic idea behind such tests is that a systematic error is caused by the upstream sequence within a read, and thus should not occur in reads approaching the position from the opposite direction. Indeed, a lack of correlation between error rates in forward and reverse reads has been noted [71], prompting independent analysis of true variant probability in forward and reverse reads [71,72]. Observed strand bias can also be utilised directly to test the validity of a variant [66,73]. For example, the latest version of ShoRAH (http://www.bsse.ethz.ch/cbg/software/shorah) incorporates a beta-binomial test of strand bias when calling variants from reconstructed haplotypes, while LoFreq implements a two tailed Fisher’s exact test. [73] A recent comparison between several of these variant calling algorithms revealed that for samples where errors are random and true diversity is high (i.e. two or more true SNVs can be expected to occur within an observed read) probabilistic clustering is a powerful technique allowing detection of SNVs at frequencies lower than the corresponding sequencing error rate. When these conditions are not met, then a statistical test of strand bias improves the precision of SNV calling [66].

The systematic nature of sequencing errors is also utilised in the ‘matched samples’ approach. Developed initially for deep sequencing of cancer cells, matched control (e.g. normal tissue) and test (e.g. tumor tissue) samples are sequenced simultaneously, ideally being multiplexed together to minimise between sample error profile variability. The error profile of the control sample is then used to account for errors in the test sample, by statistically comparing the population frequency of each potential variant in the two samples [72]. Such an approach is also feasible for pathogen deep sequencing, provided suitable controls are available. In fact, a related approach has been developed directly for viral deep sequencing, where multiple reference samples are multiplexed together with test samples, and used to calculate error rates, which then inform a statistical test [71]. This method performed extremely well, identifying a known antiviral resistance mutation with a population frequency of just 0.18% in a clinical H1N1 influenza A sample [71]. However, matched sample sequencing may be inaccessible in some cases due to sequencing costs and the requirement for suitable control samples. Also, while it can be used successfully in situations where clustering or phasing approaches may fail (for example, low diversity populations), for high diversity data it does not facilitate haplotype reconstruction *per se,* a distinct advantage of the clustering or co-occurrence approaches used by ShoRAH and V-phaser, respectively.

The statistical matched samples methods described above are essentially empirical; an alternative empirical method allowing for haploytpe reconstruction and not requiring a control sample has been implement for amplicon resequencing. This approach uses two algorithms, k-mer based error correction (KEC) and empirical frequency threshold (ET) [74]. KEC first calculates the frequency of k-mers for all reads in a data set. A frequency threshold is then estimated, with low frequency k-mers assumed to be errors. Locations within reads containing these low frequency k-mers are then subjected to error correction. The ET algorithm is a detailed multistep process for correction of homopolymers, relying heavily on accurate pairwise alignment of reads against a set of known reference sequences and error threshold estimates. Benchmarking using known amplicon sequences suggested these empirical algorithms find as many true haplotypes as probabilistic clustering, with fewer false positive haplotypes detected.

As described above, several algorithms have been proposed for haplotype reconstruction, although a benchmark comparison between these methods is still lacking (see also [75] for a review of the current methods for haplotype reconstruction). Most algorithms involve some form of local error correction, followed by a clustering or grouping step. In some cases, the option to reconstruct long haplotypes by considering overlapping short haplotypes (or reads) is provided.

Avoiding the formation of chimeras is a key challenge in haplotype reconstruction, in addition to the error correction methods discussed above. Chimeras occurring within a read and resulting from recombination during library preparation may be minimised by choosing an amplification protocol with minimal cycle numbers or stringent amplification conditions. Algorithms for removing PCR chimeras are also in development; for instance, the Perseus algorithm within the program AmpliconNoise [76] identifies chimeras by harnessing the fact that both parents of a PCR chimera must have at least one more amplification round than the chimera. Pairwise alignments between a read and its possible parents combined with consideration of their individual frequencies are used to flag potential chimeras, which are then classified using a parsimony informed supervised learning approach.

The best way to avoid *in silico* chimeras is to restrict haplotype analysis to individual read lengths. This will be increasingly feasible as NGS read lengths improve. Additionally, reduction in error rates, and improved methods for error detection and removal (see discussion on variant calling above) will be of assistance, by reducing misleading paths caused by retained errors, and decreasing the effects of false positive SNVs on haplotype diversity measures.

Finally, independent of the method used for calling variants, manual inspection of results is standard practice. For example, Bull *et al.* excluded viral variants as errors if manual inspection demonstrated that variants were only present in the ends of reads, or adjacent to a homopolymer region [14].

## Conclusions and future directions

Deep sequencing has the potential to revolutionise our understanding of pathogen evolution, providing unprecedented, real time insights into the genetic diversity of pathogen populations. Through careful experimental design and the use of appropriate controls, biases and errors can be minimised. Bioinformatic methods for separating true variants from errors are developing rapidly, and already allow detection of variants with population frequencies under 1%.

In general, all variant detection approaches discussed in this review are largely focussed on the analysis of SNVs. However, indels are also likely to contribute to pathogen evolution and therefore methods for their detection in the presence of sequencing error should receive urgent attention. Automated tools for deep sequencing analysis of bacterial populations are also required. As new sequencing technologies continue to emerge, other future bioinformatic challenges will include developing algorithms for aligning very long reads, and coping with the unique error profiles of each sequencing technology.

## Abbreviations

NGS: Next generation DNA sequencing; HCV: Hepatitis C virus; SNV: Single nucleotide variant; RT: Reverse-transcriptase.

## Competing interests

The authors declare that they have no competing interests.

## Authors’ contributions

All authors contributed to the conception and writing of the review article. All authors have read and approved the final manuscript.
